# Non-Systemic Drugs: A Critical Review

**DOI:** 10.2174/138161212799504858

**Published:** 2012-04

**Authors:** Dominique Charmot

**Affiliations:** Ardelyx, Inc., 34175 Ardenwood Blvd, Fremont, CA 94555, USA

**Keywords:** Non-systemic drugs, non-absorbed drugs, gastrointestinal tract, absorption, tight-junctions.

## Abstract

Non-systemic drugs act within the intestinal lumen without reaching the systemic circulation. The first generation included polymeric resins that sequester phosphate ions, potassium ions, or bile acids for the treatment of electrolyte imbalances or hypercholesteremia. The field has evolved towards non-absorbable small molecules or peptides targeting luminal enzymes or transporters for the treatment of mineral metabolism disorders, diabetes, gastrointestinal (GI) disorders, and enteric infections. From a drug design and development perspective, non-systemic agents offer novel opportunities to address unmet medical needs while minimizing toxicity risks, but also present new challenges, including developing a better understanding and control of non-transcellular leakage pathways into the systemic circulation. The pharmacokinetic-pharmacodynamic relationship of drugs acting in the GI tract can be complex due to the variability of intestinal transit, interaction with chyme, and the complex environment of the surface epithelia. We review the main classes of non-absorbable agents at various stages of development, and their therapeutic potential and limitations. The rapid progress in the identification of intestinal receptors and transporters, their functional characterization and role in metabolic and inflammatory disorders, will undoubtedly renew interest in the development of novel, safe, non-systemic therapeutics.

## 1 INTRODUCTION

Non-absorbable drugs are a unique subset of orally administered agents that exert their therapeutic effects locally in the GI tract. In contrast to traditional drugs, which are designed to be rapidly absorbed, to achieve therapeutic plasma levels, and then be eliminated by multiple pathways, non-absorbable drugs are designed to minimize systemic exposure. Because of these fundamental differences, the discovery and development of non-absorbable drugs is directed by its own unique and still-developing set of pharmaceutical design principles, which will be described in this review. We will also provide examples of the broad utility of non-absorbable drugs, which are used extensively to treat systemic metabolic and mineral disorders as well as their more obvious applications to diseases of the GI tract.

Early examples of non-absorbable drugs were relatively unsophisticated cation and anion exchange resins that remove bile acids or potassium ions from the lumen of the gut for the treatment of hypercholestemia and hyperkalemia, respectively. These resins were grandfathered in by the Food and Drug Administration (FDA) in the 1960s and are still in use today. Building on these early successes, the development of non-absorbable drugs has expanded greatly and now encompasses a variety of agents, polymers, small molecules, or peptides, acting throughout the GI tract and providing therapeutic benefit across a wide array of indications. In some examples the non-absorption characteristics were found serendipitously, while other drugs were rationally designed for that purpose. A misperception about non-systemic drugs is that, because they are confined to the GI tract, they can only treat GI disorders: we describe in this review that the majority of uses are for treatment of systemic diseases such as mineral or metabolic disorders. With safety criteria becoming increasingly important and approval criteria becoming more stringent, particularly for the treatment of chronic disease in large patient populations, non-systemic drugs offer the great advantage of minimizing off-target systemic effects and thereby greatly reducing risks of drug-drug interaction, toxicity, or side-effects. As a result the field has registered a number of innovative products at various stages of development using increasingly sophisticated approaches.

The terms *non-systemic *and *non-absorbed *have been used interchangeably for drugs of very different absorption profiles and metabolism pathways, and we will use the same terms throughout this review. We first briefly review the main mechanisms of drug absorption and drug disposition of non-absorbed drugs. We then review existing and emerging non-systemic drugs according to their mode of action, targets, and therapeutic actions.

## 2 CLASSIFICATION OF NON-ABSORBED DRUGS

In addition to being extremely diverse in nature and structure, non-absorbed drugs act therapeutically via various modes. We propose allocating non-systemic drugs to five classes characterized by common attributes schematically represented in Fig. (**1**), as follows:

- **Sequestering agents:** their primary function is to bind a small molecule such as a nutrient, an endogenous ligand or a toxin in the lumen of the gut to form an insoluble complex ultimately eliminated in the feces.
- **Ligands of soluble intestinale nzymes:** these compounds target host proteins resident in the gut lumen: Representatives of this class are small molecules that inhibit the activity of digestive enzymes (*e.g.* lipase, saccharidase) there by blocking the digestion of dietary components into readily absorbable entities (*e.g.* triglycerides into fatty acids; dietary polysaccharides into glucose, fructose and galactose), resulting in a netdecrease in nutrient absorption.
- **Enzymes:** either of animal origin or recombinant proteins, acting in the gut lumen to compensate for an enzyme deficiency in the host or to metabolize certain bacterial or metabolic toxins; an example of the former is the treatment of cystic fibrosis induced pancreatic insufficiency by administration of porcine pancreatic enzymes.
- **Minimally absorbed and rapidly metabolized:** these drugs are locally absorbed and act on targets expressed within the inner wall of the gut (lamina propria or myenteric muscles). The parent drug is quickly degraded by first-pass metabolism in the enterocytes and/or in the liver, with spurious low levels in systemic circulation. This class of drugs (also referred to as *soft drugs*) is designed to undergo rapid cleavage or conjugation by first- and second-pass metabolism, and has been reviewed elsewhere [1-4]
- **Ligands of apical targets:** these agents specifically target membrane proteins expressed on the surface of the gut epithelia. These compounds are non-absorbed synthetic molecules or peptides fulfilling a variety of functions, including inhibiting transporters and channels, binding to G-Protein coupled-receptors (agonists and antagonists), or modulating tight junction (TJ) permeability. This class will undoubtedly expand given the myriad of potential targets and their potential role across many therapeutic areas.

## 3 FACTORS GOVERNING GUT PERMEABILITY TO DRUGS

The intestinal tract is endowed with a multitude of absorption and sampling pathways to selectively and actively transport nutrients from the lumen of the gut into the bloodstream. It is has a combination of passive and active transport, and efflux mechanisms operating at the enterocyte level [5], while other pathways involve M cells and dendritic cells sampling luminal entities via endocytosis / pinocytosis. The absorption of systemic drugs generally proceeds by passive or active transport within the enterocytes as well as by passive paracellular transport through the TJs. Many phenomenological approaches and attempts at modeling drug absorption in the GI tract have been reported; the most popular one is referred to as the Lipinski’s rule [6]. Such methods are now completely integrated in drug discovery and lead optimization methods, providing medicinal and computational chemists valuable semi-empiric tools to predict the absorption of small molecules drugs. For instance Lipinski established a combination of criteria for molecular weight, lipophilicity, hydrogen donor-acceptors, and rotatable bonds (*Lipinski’s Rule of Five*, or *Lipinski’s Rule*) that is judged essential for gut absorption. In this heuristic, a compound is more likely to be efficiently absorbed from the GI tract if at least three of the following are true:

- The molecular weight is not more than 500daltons
- There are five or fewer hydrogen bond donor groups
- There are ten or fewer hydrogen bond acceptor groups (generally nitrogens and oxygens)
- The octanol water partition coefficient is not greater than 10^5^

Alternative heuristics have gained acceptance among chemists, such as the one based on the computation of total polar surface area (tPSA) [7,8]. Many of these methods can be viewed as alternative formulations of the Lipinski criteria; for example, the tPSA of a compound is a function of its molecular weight and the number of hydrogen bond donors and acceptors. These computational methods are generally applicable only to drugs that are passively absorbed by the transcellular route (see below). Hybrid models combining empirical and computational methods are also used [9].

The gut permeability profile is variable among individuals; it varies with age and is subject to alterations due to pathological situations, such as inflammatory bowel disease (IBD), various genetic diseases, and irritable bowel syndrome (IBS). In that context, the notion of a strictly non-absorbed drug is elusive; a finite fraction of the drug, as parent compound, metabolites, or impurities will inevitably be absorbed and disposed of systemically; notwithstanding all efforts to minimize drug intake, only empirical pharmacology and toxicology methods will determine whether the drug is safe and well tolerated.

## 4 NON-ABSORPTION CRITERIA ACROSS CLASSES OF NON-ABSORBABLE DRUGS

The criteria used to characterize the pharmacokinetic profile of a non-absorbed drug are essentially the same as those used for the absorption, distribution, metabolism, and excretion (ADME) profile of bioavailable drugs, and consist of establishing drug and metabolite levels in arterial blood, the portal vein, urine, and feces, as well as determining the tissue distribution.

### 4.1 Sequestering Agents

Sequestering agents, also referred to as binding resins, are usually synthesized as crosslinked polymeric particles of various size and shape. When in contact with intestinal fluid, the resin swells allowing a reciprocal transfer of endogenous ions (K^+^, HPO_3_^2-^, or bile acids) with the counter ions contained in the resin (Na^+^ or Cl^-^ depending on whether the resin is a cation or anion exchange material). These polymers are resistant to gut metabolism and are mostly recovered intact in the feces.

The systemic absorption of sequestering agents is minimized by controlling particle size and by minimizing the presence of soluble impurities. It is generally agreed that intestinal uptake of particles correlates negatively with particle diameter. Maximal absorption occurs with particles of 50 to 100 nm in diameter, while particles larger than 1 µm are trapped in the Peyer’s patches and do not translocate to systemic circulation [10]; manufacturing processes are controlled to minimize the presence of particles in this size range. Soluble impurities mostly comprise oligomers entrapped within the polymer matrix, or degradation products. Because sequestering agents (*e.g.* phosphate and bile acid binders) are typically dosed between 2 and10 g/day, a substantial quantity of impurities could potentially be released into the luminal space; therefore washing polymers thoroughly to achieve a specified water soluble oligomer content (typically less than 0.1 weight%) is a necessary and often costly aspect of polymeric drug manufacturing processes. The methodology for characterizing plasma drug content is irrelevant as resins rarely appear as such in blood circulation and are not easily characterizable as a drug entity because of their very large molecular weight and polydisperse nature; more typically impurities and degradation products are first identified in stress-stability testing or *in vitro* metabolism studies and then monitored in plasma. However, the gold standard method remains radioactive labeling with^14^C and measuring the distribution of the label in urine, feces, and body tissues; typically synthetic crosslinked resins are almost quantitatively recovered in the feces, with yields ranging from 82% [11] to 99% [12]. Certain sequestering materials are made of insoluble metallic inorganic materials that release soluble metallic salts (Fe, Mg, La), which can potentially be absorbed. Therefore accumulation of those metals in plasma and tissues is closely monitored.

### 4.2 Non-Absorbed Small Molecules

Most of the agents described in classes ii and iv are small molecules. Their non-systemic profile is conveniently characterized by traditional drug metabolism pharmacokinetic methods, tracking parent drug and metabolites in blood, urine, and feces. Low or undetectable levels of drug and metabolites in serum do not necessarily imply non-absorption; the portal vein and bile should be assayed for the presence of drug and metabolites to check for gut absorption followed by hepatic uptake and disposition in bile. Loperamide, an antidiarrheal agent with very low plasma levels, is extensively effluxed from the enterocytes but is nevertheless present in the intestinal wall [13] and partly eliminated from systemic circulation via hepatic uptake and excretion virtually intact in the feces. The preclinical transport findings for enterocyte efflux mechanisms may not translate to a non-systemic profile in humans because of species differences in transporter expression, substrate affinity, physiological function, and interplay between transporters and enzymes [14]. Ideally a non-systemic drug should be virtually absent in blood and urine, and the parent + metabolites should be recovered quantitatively in the feces. In reality, non-systemic drugs have a low but finite exposure in systemic compartments, from single to double digits ng/mL. Fecal recovery varies widely, from 0% for exogenous peptides or protein (due to extensive proteolytic degradation by host and bacterial proteases) up to 97% for the non-systemic antibiotic rifaximin [15]. As evidenced by radiolabel ADME studies [16], even in favorable cases mass balance studies often result in less than 100% recovery due to experimental limitations.

Historically, non-absorbable small molecule therapeutics were designed to selectively hit an intestinal target, but were not specifically engineered to be impermeable to the gut epithelia. Many of these compounds were discovered serendipitously by screening natural products, as was the case for or list at (lipase inhibitor), acarbose (α-glucosidase inhibitor), and several antibiotics (oral vancomycin, rifaximin) of bacterial or fungal origin. The structural requirements imposed on small molecules to prevent diffusion across the intestinal lining, are clearly different and much more stringent than those of large crosslinked polymer particles physically segregated to the intestinal lumen. Small molecules have dimensions similar to those of substrates involved in molecular uptake mechanisms (transporters and efflux pumps), and those of pores within TJs.

Lipinski’s rule was designed to be predictive of solute passive transport through the lipid bilayer of enterocytes; designing compounds outside of Lipinski’s rule would prevent transcellular transport. However, these rules do not address the passive diffusion pathway through the TJs or transporter-mediated absorption. Therefore, the more stringent criteria for non-systemic drugs requires not only designing molecular structures outside of the Lipinski’s rule but also avoiding structures that are recognized by intestinal transportersor subject to passive diffusion through the TJs.

Recent progress in identifying the diffusion mechanisms through the TJs has provided some insight about solute requirements for permeation. Tight junction permeability experiments have revealed a high capacity pathway for solutes less than about 0.4 nm in radius [17] mediated by claudin proteins, and a second pathway, permeable to molecules with molecular weights up to several kg/mol. The latter pathway is due to time-dependent openings in the otherwise continuous mesh of adherent TJ proteins (claudins, occludins, and junction-adhesion-molecules) [18]. In addition to the maximum size threshold imposed by the TJ pore distribution, it appears that the charge density and rigidity of the solute (here the drug molecule) tightly regulate passage across TJs. This area has been thoroughly investigated for enhancing TJ permeability to therapeutic peptides [19]. A survey of the mechanisms involved is out of the scope of this review. We note only that permeability does not necessarily correlate with molecular weight, and seems to better correlate with molecular cross-sectional diameter, or volume. Intuitively solute charge density might be thought to play a role in TJ permeation; however, recent studies with polypeptides of controlled charge density concluded it is unimportant [19].

Taken in the context of non-systemic drugs, this would imply that molecules with high charge density and low flexibility would have low TJ permeability, and therefore limited paracellular diffusion.

The exact nature of the paracellular diffusion has yet to be elucidated and more research is required to establish precise structural requirements for non-systemic molecules. The field is nascent and the collection of non-systemic drugs too limited to train a Lipinski-like heuristic model for non-systemic drugs. General recommendations can be made based on the emerging knowledge on TJ transport mechanisms; however, only an experimentally based approach will guide the scientist effectively.

## 5 PHARMACOKINETIC-PHARMACODYNAMIC RELATIONSHIP IN NON-ABSORBED DRUGS

The pharmacokinetic-pharmacodynamic relationship is the “temporal aspects of drug action, and particularly the relationship between the concentration of drugs (and their active metabolites) in plasma or other biological fluids and their intensity and time course of their pharmacological effects” [20]. For non-systemic drugs, *other biological fluid* refers to the intestinal lumen, a body compartment rarely explored in drug development. However understanding the pharmacokinetic-pharmacodynamic relationship is critical for optimizing drug performance, dosage regimen, and formulation. For example, agents blocking absorption or digestion of dietary components must be bioavailable in the upper part of the gut and provide optimal exposure to the gut target, whether apical or luminal. As opposed to systemic targets continuously perfused by biological fluids and drug at relatively constant composition, the lumen of gut is highly heterogeneous with pockets of fluids revealed by magnetic resonance imaging (MRI) techniques [21] that show rapid changes in luminal *free water *as a function of diet composition or bowel disorders [22]. For non-systemic small molecule drugs, interaction with chyme components can reduce drug availability and potency [23], a situation analogous to that of systemic drugs binding to plasma proteins. Finally the role of the mucus layer on drug transient accessibility to the epithelia surface remains poorly understood.

## 6 THERAPEUTIC APPLICATIONS OF NON-SYSTEMIC DRUGS

### 6.1 Mineral Metabolism Disorders

#### 6.1.1 Phosphate Lowering Drugs

Patients with declining kidney function progressively lose the ability to excrete phosphate renally. The ensuing positive phosphorus balance triggers a hormonal response via FGF-23, parathyroid hormone (PTH) and active vitamin D. High-normal serum phosphorus elevates PTH which in turn induces bone resorption, causing phosphorus release, thus creating a vicious cycle resulting in secondary hyperparathyroidism. This hormonal imbalance and concomitant phosphorus overload result in an increased risk of vascular calcification, a risk factor for cardiovascular events, which are highly prevalent in chronic kidney disease (CKD) patients.

The first phosphate binder was aluminum carbonate, which sequesters dietary phosphate in the gut as insoluble aluminum phosphate salts. Because of its toxicity, aluminum was later replaced by calcium (acetate and carbonate salts), now generic drugs used in phosphate management for CKD. However, systemic absorption of calcium has fueled a controversy on the use of those agents, as vascular calcification is major risk for CKD patients. Sevelamer (RenaGel®; Genzyme-Sanofi), the first non-metallic based phosphate binder, was introduced to the market in 2000 for the normalization of serum phosphorus in dialysis patients. It is a crosslinked polyallylamine gel, binding phosphate via a combination of electrostatic and hydrogen bond interactions. The binding efficacy is approximately 0.5 mmol/g of polymer as calculated from a phosphorus balance study in humans [24]. Sevelamer also binds bile acids, thus reducing low-density lipoprotein (LDL) cholesterol levels via induction of bile acid biosynthesis from the cholesterol pool. Other phosphate binding agents based on synthetic polymers or inorganic salts (Mg, Fe) have been developed [25] in an effort to improve the selectivity and capacity with a goal of reducing the dose burden, currently the main obstacle to patient compliance. Bixalomer (Amgen/Astellas) is a next generation phosphate binder composed of microspheres of a crosslinked polyamine polymer, with a pore size optimized to selectively bind phosphate while minimizing fatty acid and bile acid uptake [26,27].

The suboptimal clinical efficacy of phosphate binders is caused in part by competition from active intestinal phosphate transport. A radically different approach involves the development of phosphate transport inhibitors targeting the intestinal sodium/phosphate co-transporter NaP2b (SLC34A3). NaP2b is expressed throughout the upper GI tract and mediates about half of dietary phosphate uptake [28]. Because NaP2b is expressed in other tissues, it is imperative that the inhibitor be non-absorbed and maintained within the intestinal lumen. In one report, non-systemic NaP2b inhibitors (Amgen) were shown to reduce gut absorption of phosphate in rats [29].In another study, non-absorbable NaP2b inhibitors (Ardelyx) used alone or in combination with a phosphate binder were evaluated in two animal models: in an adenine-induced CKD rat model, NTX1942 reduced uremic biomarkers (serum concentration of phosphorus, creatinine and BUN), as well as parathyroid hormone and FGF-23 plasma levels; in a 5/6^th^ nephrectomy rat model, it delayed deterioration of renal function and, after 50 days of treatment, showed a significant increase in survival outcome (Gehan-Breslow-Wilcoxon curve comparisons) compared to vehicle-treated cohort [30]. Although those results are very preliminary they are consistent with the prominent role of body phosphorus in the elevation of FGF-23 and its causal role in the pathogenesis of left ventricle hypertrophy contributing directly to high rates of cardiovascular mortality in individuals with CKD [31]. As the clinical dose would likely be substantially lower than that of phosphate binders, this new class of phosphate lowering drugs could provide a much needed phosphate reduction in the broader pre-dialysis CKD patient population.

#### 6.1.2 Potassium Lowering Drugs

Hyperkalemia is characterized by serum potassium higher than 5.5 mmol/L, and can be an acute life-threatening condition if not quickly resolved. Hyperkalemia is common in CKD patients as their urinary potassium excretion is impaired. The risk is notably increased with the use of renin-angiotensin-aldosterone-system (RAAS) blockade drugs to treat hypertension and/or heart failure. Angiotensin-converting enzyme inhibitors, angiotensin receptor blockers and mineralocorticoid receptor antagonists all reduce renal sodium reabsorption via down regulation of the epithelial sodium channels (ENaCs) with a concomitant potassium-sparing effect leading to chronic hyperkalemia. Non-absorbed potassium binding drugs have long been used to treat acute hyperkalemia; they consist of sulfonated crosslinked resins neutralized with either sodium ions (Kayexalate®) or calcium ions (Sorbisterit®, Ca-Resonium®, Argamate®). They act as cation exchangers substituting Na^+^ or Ca^2+^ for K^+^ in the lower GI tract, thereby draining potassium from systemic circulation. By binding potassium in the lumen of the colon, those polymers create a steeper K^+ ^electrochemical gradient at the colonic epithelia, enhancing mucosal transport via potassium channels expressed apically at the mucosal surface [32,33]. These resins were not originally designed as non-absorbable drugs but were merely re-formulated from existing industrial grade cation-exchange resins. They present as irregularly shaped particles, mixed with sorbitol, an osmotic laxative, to favor transit and mitigate constipation. Cases of intestinal necrosis, which may be fatal, and other serious gastrointestinal adverse events (bleeding, ischemic colitis, perforation) have been reported in association with Kayexalate use [34]. The reason for those serious GI side-effects is unclear, but one could invoke the irregular shape of the particles that could induce micro-laceration of the mucosa, and also the particle suspension shear-thickening flow behavior leading to impaction. The main shortcomings of these resins are their modest *in vivo *binding capacity and selectivity. The patient is thus required to take dozens of grams per day, causing many GI side effects for a modest benefit. Recently a crosslinked poly-α-fluoroacrylic acid polymer calcium salt with higher capacity and better physical properties [35,36] was developed to improve efficacy and patient compliance. Super-absorbent materials based on crosslinked polyacrylic acid to bind either potassium or sodium by adjusting the Na^+^/K^+^/H^+^cation composition have also been proposed; the resin is designed to swell with luminal fluid so is also capable of removing fluids in patients with fluid overload [37]. Cation binding selectivity remains a challenge since polymeric anion-exchangers have greater affinity for divalent cations (Ca^2+^, Mg^2+^) than for monovalent cations (Na^+^, K^+^), thus greatly reducing the potassium binding efficiency in the colon. For that reason, composite particles were designed, comprising a cation-exchange polymer particle (crosslinked poly-α-fluoroacrylate) with the surface covered by a thin pellicle of a second polymer (crosslinked polyvinylamine). The *shell* polymer is permeable to monovalent ions such as K^+^, but impermeable to divalent cations (Ca^2+^). Such *core-shell* particles increase the *in vivo* binding capacity for potassium compared to regular non-core-shell resins [38].

#### 6.1.3 Treatment of Sodium Overload

Fluid and sodium overload are symptomatic of hypertension, congestive heart failure (CHF), and CKD. Insufficient cardiac output and/or kidney failure triggers activation of RAAS, which then cascades into hypertension and cardiovascular and renal damage. Hypertension is tightly related to sodium homeostasis; decades of clinical trials have demonstrated the link between sodium intake and blood pressure. A 3 g/day reduction in salt intake decreases systolic and diastolic pressure by 5 and 2 mm Hg, respectively, leading to notable reductions in hypertension-related morbidity and mortality.

More than 80% of CKD patients are hypertensive and salt-sensitive, aggravating the decline of kidney function. To manage their hypertension CKD patients are given ever increasing doses and combinations of RAAS inhibitors and diuretics. As kidney function declines, thiazide diuretics lose efficacy causing physicians to treat with increasing doses of loop diuretics, ultimately resulting in an acceleration of kidney function decline. Moreover, excess dietary sodium limits the benefit of RAAS blockade drugs [39]. Sodium overload is also a hallmark of CHF patients with concomitant renal impairment, *i.e. *cardiorenal syndrome.

There are almost no literature reports of non-systemic agents that alleviate sodium overload by sequestering sodium in the gut. A few studies in the 1950s tested the ability of cation-exchange resins to resorb edema in heart failure patients [40,41]. The challenge is practically insurmountable given: i) the amount of sodium to remove coupled with the modest *in vivo* binding capacity of regular cation-exchange resins at colonic pH; ii) the release of one equivalent of K^+^ or Ca^2+^for every bound Na^+^; and iii) the rapid cation exchange of Na^+^ for K^+^ occurring in the colon as a result of the greater concentration of K^+^ in the lower GI tract (*vide supra).*

Recognizing that the main pathway for sodium re-import present throughout the GI tract is the Na/H antiporter 3 (NHE3) has led to the development of highly potent and non-systemic NHE3 inhibitors as an alternative approach to managing sodium overload. Representative of this class is RDX5791, a molecule that modulates the uptake of intestinal sodium from dietary or endogenous sources. Preclinical data in the rat indicate that NHE3 blockade shifts sodium almost entirely from urinary to fecal excretion. To further validate its therapeutic use in the setting of hypertension in CKD, RDX5791 was tested in five-sixths nephrectomized rats fed a high sodium diet. NHE3 inhibition reduced blood pressure, normalized albuminuria, and delayed heart and kidney damage [42].

#### 6.1.4 Iron Overload

In hemochromatosis patients, abnormal dietary iron absorption leads to iron deposition in vital organs, including the liver, endocrine glands, heart, and skin. The most common form is hereditary and is due to mutation of the HFE gene that controls intestinal iron transport proteins (ferroportin and hepcidin). Treatment consists of twice weekly phlebotomy. A typical diet contains 10 to 20 mg of iron, of which only 1 to 2 mg are absorbed as non-heme iron (*i.e.* not complexed with heme component of hemoglobin) and heme-iron. The standard of care employs systemic iron chelators, injectable (deferoxamine; Desferal®) or orally administered (deferasirox; Exjade®). However, these compounds are associated with either a narrow therapeutic window (deferoxamine) or pose a risk of fatal renal failure and cytopenia (deferasirox). This provides a rationale for developing non-systemic gut-acting iron chelators. These are derived from structures typical of siderophores (hexadentate chelates of catecholamide, hydroxamate, hydroxypyridonate, 2,3-dihydroxyterephthalamide) that are made insoluble by functionalization onto polymeric backbones [43]. However, there are few examples of non-systemic iron chelators demonstrating an effect in preclinical iron overload models [44] and no clinical development has been reported. One challenge encountered by this approach is the multiple pathways utilized by the intestinal mucosa to extract dietary iron, one of which is the selective transport of heme-iron [45]. This perhaps calls for a multivalent polymeric drug with dual binding sites specific to heme and non-heme iron.

### 6.2 Non-Systemic Drugs for Treatment of Metabolic Diseases

This section encompasses non-systemic approaches to limit or delay absorption of nutrients (via inhibition of gastric lipase, α-glycosidase), cholesterol or bile acids (via inhibition of Niemann-Pick C1-Like 1 [NPC1] or Ileal bile acid transporter [IBAT], or binding of intestinal bile acid), or altering energy utilization (via inhibition of pancreatic phospholipase A2 [PLA2]).

#### 6.2.1 Pancreatic Lipase Inhibitors

Orlistat (Xenical®) isa potent pancreatic lipase inhibitor isolated from the bacterium *Streptomyces toxytricini*; it partially inhibits the conversion of dietary fat into fatty acids, thereby reducing fat absorption as only fatty acids are systemically absorbed from the GI tract. The undigested fat is eliminated in the feces.

Orlistat is partially metabolized in the gut wall with two metabolites appearing in plasma at 26 and 108 ng/mL. Eighty three percent of the parent compound (97% of the parent + metabolites) is found in the feces [46]. In clinical trials, obese patients treated with orlistat in addition to lifestyle modifications lost a modest 2 to 3 kg more than those treated with placebo [47]. The main side effect was steatorrhea (oily stools). Alizyme / Takeda are developing a close analog of orlistat, cetilistat, claimed to elicit fewer episodes of steatorrhea. Non-systemic polymeric lipase inhibitors were developed by GelTex Pharmaceuticals [48], to be combined with *fat-scavenging polymers *[49], *i.e.* lightly crosslinked polyacrylate copolymers with cationic and hydrophobic moieties. Such polymers would either bind or adsorb at the surface of undigested fat droplets to prevent coalescence, thus minimizing steatorrhea.

#### 6.2.2 α-Glucosidase Inhibitors

Acarbose (Precose®) is a complex oligosaccharide obtained through fermentation by *Actinoplanesutahensis*. It is an inhibitor of pancreatic alpha-amylase and membrane-bound intestinal alpha-glucoside hydrolase enzymes. Taken with a meal, it partially inhibits digestion of dietary polysaccharides into glucose, therefore delaying glucose absorption and blunting post-prandial serum glucose excursion. Prescribed in pre-diabetic patients, itreduces the level of glycated hemoglobin A1c (HbA1c), a measure of sustained hyperglycemia. A radiolabel study demonstrated that about 35% of the drug is absorbed while only 2% of the parent compound is found in serum. Acarbose is metabolized within the GI tract, mostly by intestinal flora. In addition to its effect on post-prandial glycemia, acarbose also seems to induce GLP-1 secretion via a glucose-mediated stimulation of entero-endocrine cells distally in the gut, potentially contributing to its anti-diabetic effect. The downside of delayed glucose absorption is frequent GI side effects arising from colonic fermentation of glucose.

#### 6.2.3 Cholesterol Transport Inhibitors

Ezetimibe (Zetia®) inhibits intestinal cholesterol absorption, leading to upregulation of LDL-receptors on cell surfaces and increased LDL-cholesterol uptake into cells, thus decreasing the plasma levels of LDL, a biomarker of atherosclerosis and cardiovascular events. The target of ezetimibeis NPC1, [50], a protein apically expressed in the upper GI tract characterized as a transporter of sterols including cholesterol [51], and probably other sterol-based metabolites such as vitamin D3 [52]. Ezetimibe is absorbed and extensively glucuronidated to ezetimibe-glucuronide, which has greater activity than the parent compound. Both the parent and its glucuronide adduct are excreted in the bile and undergo extensive entero-hepatic cycling upon de-glucuronidation and re-absorption. According to criteria presented above in *Classification of Non-Absorbed Drugs, *Ezetimibe does not strictly meet the criteria of a non-systemic drug since the parent drug and its main metabolite are present in plasma at substantial levels and likely act on hepatic NPC1 localized on the bile canalicular membrane. Whether ezetimibe may induce mechanism-based side effects due to its role in cholesterol homeostasis in the liver is unclear; however, the clinical safety profile of ezetimibe appears adequate. Several companies have been working to develop second-generation non-systemic NPC1 inhibitors by creating isosteric analogs of glucuronidatedezetimibe, substituting the O-glycoside moiety with C-glycoside or aglycon groups resistant to glycosidases and polar enough to prevent drug absorption [53-59]. In general this new class of NPC1 inhibitors achieves excellent potency against their target (nM range) with low systemic exposure; however, the clinical data are disappointing. As of November 2011, AstraZeneca and Sanofi-Aventis reported suboptimal efficacy compared with ezetimibe and abandoned further development [55,56] . Kotobuki Pharmaceutical Co is still conducting phase II studies in the United Kingdom. It is possible that the lower efficacy results from a lack of target exposure during postprandial conditions, in contrast with ezetimibe which has the great advantage of being constantly recycled at the mucosa surface via entero-hepatic cycling.

#### 6.2.4 Bile Acid Binders

The first generation of bile acid binders is embodied by cholestyramine, a crosslinked polystyrene resin functionalized with quaternary ammonium groups. It sequesters bile acids in the GI tract and up-regulates bile acid biosynthesis from circulating cholesterol, lowering plasma LDL-cholesterol. To achieve significant LDL-cholesterol reduction, 5 to 25 g of resin per day is needed, clearly limiting patient compliance. In the distal segment of the ileum, bile acids are avidly re-absorbed by the IBAT, thus limiting the efficacy of bile acid resins, a situation analogous to that of phosphate binders competing with the NaP2b phosphate transporter. Researchers at GelTex / Genzyme rationally designed terpolymers with a careful balance of cationic and hydrophobic groups to increase affinity to bile acids [60]; as important as binding capacity is that the polymer retain bile acids past the ileal segment, where free bile acids drop drastically as a result of IBAT-mediated bile acid re-uptake. The resulting drug, colesevelam (Welchol®), is significantly more potent than cholestyramine in lowering LDL-cholesterol. Anecdotally, sevelamer, a phosphate binder composed of a hydrophilic crosslinked polyaliphatic amine, has an LDL lowering capacity close to colesevelam’s, which could be easily explained by the co-binding of fatty acids and bile acids forming a tight complex [61]. In clinical studies, bile acids binders such colesevelam and colestilan showed a modest but significant diminution of HbA1c, stimulating a debate on the origin of the effect [62]. A plausible mechanism is that bile acids leak from the complex polymer / bile acid and induce GLP-1 secretion via stimulation of TGR5 receptors in colonic L cells.

#### 6.2.5 IlealBile Acid Transport Inhibitors

To achieve drastic LDL-reductions in people at risk for cardiovascular events, such as type 2 diabetic patients, high doses of statins (HMG-CoA reductase inhibitors) must be administered; however, the mechanism-based toxicity of statins (myopathy and rhabdomyolysis) strongly limits this approach. Combination approaches using orthogonal mechanisms to maintain LDL under guideline levels are preferred. In this context, IBAT inhibitors were actively pursued in the 1990s as an alternative to bile acid binders with the goal of reducing the dose and circumventing vitamin mal absorption, a common side-effect of bile acid sequestrants. IBAT inhibitors selectively inhibit the re-uptake of bile acids in the ileum, increasing fecal elimination and triggering biosynthesis of biliary salts from the cholesterol pool. Several companies worked to develop such inhibitors, and for the first time utilized a rationalized discovery effort for non-absorbable compounds. Potent molecules were developed based on bile acid pharmacophores and synthetic chemotypes including benzothiazepine, propanolamines, 4-phenyl-quinolines, and lignans, among others. The concept of *kinetophore* was introduced; a structural unit violating Lipinski’s rule was tethered to the pharmacophore moiety via a linker, making the whole molecule large and polar while maintaining potency and solubility [63].

Kinetophores were selected from short peptides, sugars, and quaternary ammonium-capped polyethylene-oxides groups. Supramolecular structures comprising two or three copies of the inhibitory units were also proposed (*i.e*. dimer and trimer of bile acid mimics). Although preclinical data were encouraging, showing significant reduction in LDL levels in hamsters, clinical data have been limited or disappointing, showing relatively modest reductions in LDL, and diarrhea side effects at doses providing the maximal LDL lowering effect. Diarrhea is not unexpected as the presence of bile acids in the colon is known to elicit a pro-motility and prosecretory effect. The modest efficacy in the presence of GI side effects has stopped all clinical development of IBAT inhibitors for the treatment of hypercholesterolemia; no clinical developments have been reported since 2001. Recently, GlaxoSmithKline showed that in a diabetic rat model, the benzothiazepine IBAT inhibitor 264W94 increases bile acids in the distal intestine, promoting the release of the endocrine peptide GLP-1 and its accompanying satiety and energy expenditure signaling, improving HbA1c and insulin signaling [64].

#### 6.2.6 Phospholipase A2 IBInhibitor

The digestion of dietary and biliary phospholipids by pancreatic phospholipase A2 IB (PLA2 IB) is considered a necessary step in cholesterol absorption; studies with PLA2 IB inhibitors or PLA2 IB knock-out mice showed a delay in cholesterol uptake [65,66]. Nevertheless compensatory mechanisms have limited the pharmacological impact of PLA2 inhibition by this approach. More recently, pharmacological or genetic blockade of PLA2 IB revealed a new potential for treatment of obesity and diabetes. Lysophospholipids produced by PLA2 IB hydrolysis suppress hepatic fat utilization and down-regulate energy expenditure [67,68]; the PLA2 IB inhibitor methyl indoxam effectively suppresses diet-induced obesity and diabetes in mice [69]. Dimeric PLA2 inhibitors with minimal bio-availability were optimized based on the methyl indoxam pharmacophore, and preclinical data on rodent models were reported in the patent literature [70].

### 6.3 Binding or Neutralizing Intestinal Toxins

#### 6.3.1 Clostridium difficile Toxin Binder

*Clostridium difficile (CD)* is a gram-positive bacterium responsible for many cases of antibiotic-associated colitis (CDAD) [66,71,72]. Toxigenic strains of *C. difficile* produce two exotoxins, toxin A and toxin B, with toxin A generally accepted as the primary toxin responsible for producing clinical symptoms [73]. It is a nosocomial disease; patients acquire the organism from hospital medical staff and the contaminated environment [74]. The major challenge in therapy is in the management of patients with multiple relapses, where antibiotic control is problematic.

Several approaches have been reported for directly neutralizingCD toxin activity in the intestinal tract using multi-gram quantities of anion exchange resins (cholestyramine, colestipol). Cholestyramine, a cationic resin that binds to *C. difficile *toxins, has been used as a treatment for *C. difficile *colitis in some patients. This resin has shown only modest activity and is not recommended for use in patients with severe cases of *C. difficile *colitis. Cholestyramine also binds to vancomycin and must be dosed separately if it is used in combination with this antibiotic. In a rat ileum CD toxin A challenge assay, cholestyramine showed weak activity compared to for instance linear high molecular weight polystyrene sulfonate, the toxin binder developed by Geltex as Tolevamer [75]. The tolevamer-CD toxin A interaction is polyvalent and of relatively high affinity, operating via a combination of electrostatic and hydrophobic forces [76].

The structures of the CD toxins have been studied extensively [77], and four functional domains have been identified. The ‘B’ domain, also known as the binding C-terminal domain, has elements that bind specific sugars on the surface of enterocytes. Toxin A avidly binds to the trisaccharide Gal(α1-3)Gal(β1-4)GlcNAc. Paradoxically, this structure is found in most mammals, but not humans. It has been proposed that in human colon, the *C. difficile* bacterium secretes α-fucosidase that converts the endogenous human sugars to the appropriate target. Whatever the mechanism, there is compelling evidence that binding these lectin-like proteins is a key first step in the endocytosis process essential for virulence.

A toxin A specific sorbent was prepared by attaching the receptor epitope (trisaccharide Gal(α1-3)Gal(β1-4)GlcNAc) to the surface of large aluminosilicate spheres. This material (synsorb) [78] was tested in patients but not pursued further because of the large dose and gritty nature of the beads. Later, the same epitope was anchored to the surface of polymeric nanoparticles. The spacing of the trisaccharide and the composition of the nanoparticle backbone were varied to enhance the polyvalent interaction and toxin neutralization activity *in vitro* and *in vivo *[79]. Despite encouraging results in early clinical trials, neither synsorb nor tolevamer toxin binders had confirmed clinical efficacy in later stage development. Oral vancomycin, a low bioavailability gram positive antibiotic, is prescribed for recurring cases of CDAD; the long-awaited next generation, non-systemic gram positive antibacterial fidaxomicin [80] is an 18-membered macrocyclic antibacterial compound and has “minimal systemic absorption” according to its package insert; its main metabolite OP-1118, the hydrolysis product of the isobutyryl ester, however, is detectable in the plasma of patients in the range 22-50 ng/ml. In clinical trials, fidaxomicin was no less effective than vancomycin in the treatment of *C. difficile* infection, based on non-inferiority analyses of clinical cure rates. The rate of recurrence, a hallmark of *C. difficile* infection, was significantly lower with fidaxomicin than with vancomycin therapy in patients infected with non-NAP1/BI/027 strains (8.4% vs 25.3%), but not significantly different in patients infected with the NAP1/BI/027 strain (23.3% vs. 31.2%) [81].

#### 6.3.2 Detoxification of Enteral Lipopolysaccharides

Only recently has the pivotal role of bacterial endotoxin been recognized in the etiology of GI diseases. An abnormally permeable gut mucosa allows slow infusion of lipopolysaccharides (LPS) from the lymph nodes and portal vein into key organs such as the liver. Mild endotoxemia due to LPS at concentrations orders of magnitude lower than those encountered in septic shock, is now hypothesized to be a risk factor in IBD [82], non-alcoholic-steatohepatitis (NASH) [83], insulin resistance [84], chronic heart failure [85], and progression of AIDS [86].

Although IBD is a multifactorial disease that cannot be reduced solely to the effect of LPS, the detoxification of luminal LPS has produced interesting results. In a small pilot clinical study, IBD patients with ulcerative colitis were treated by duodenal infusion of bovine alkaline phosphatase, an LPS dephosphorylating agent, with an encouraging resolution of symptoms [87].

#### 6.3.3 Detoxification of Gluten in the Treatment of Celiac Disease

In genetically susceptible patients, exposing the small intestine to gluten induces a CD4+ T-cell mediated inflammatory response, leading to destruction of the intestinal villous structure. The principal toxic components of wheat gluten belong to a family of closely related proline and glutamine rich proteins called gliadins [88]. Non-absorbable copolymers of hydroxyethyl methacrylate and 4-styrenesulfonate have been proposed to neutralize luminal gliadin [89], but the most advanced approach utilizes glutenases to selectively digest these immunogenic peptides in the upper gut [90]. One combination of these proteases, ALV003 (Alvine Pharmaceuticals), is currently in phase II clinical development, and preliminary results show biopsy proven clinical efficacy in celiac disease patients [91].

#### 6.3.4 Restoring Gut Integrity via Modulation of Tight Junction Permeability

AT-1001 (Alba Therapeutics) is an octapeptide derived from zonula occludens toxin (ZOT), a prokaryotic protein secreted by *Vibrio cholerae* that appears to bind to a receptor on the apical surface of enterocytes. AT-1001 inhibits gliadin-induced intestinal epithelial cell cytoskeleton rearrangement and TJ disassembly. Pre-treatment with the peptide blocks gliadin-induced leak in diseased (celiac) human duodenal epithelia [92]. In a double-blind placebo-controlled safety study in celiac disease subjects, AT-1001 was undetectable in plasma and appeared to reduce intestinal barrier dysfunction [93].

#### 6.3.5 Uremic Toxin Binders

The uremic syndrome is attributed to progressive retention of a large number of toxins, which under normal conditions are excreted by the healthy kidneys. Some of these toxins originate from the diet, and thus can be targeted by non-absorbable materials with affinity towards those toxins. Indoxyl sulfate and cresyl sulfate are toxins generated in the intestine and liver by first-pass metabolism of indole and cresol, which are formed upon digestion of dietary tryptophan and tyrosine, respectively. Indoxyl sulfate promotes progression of glomerular sclerosis and CKD *in vivo *[94-96] via activation of the NF-κB pathway, inducing cytotoxicity in renal proximal tubular cells [97]. AST-120 (Kremezin®) consists of high-surface area activated carbon particles, which given orally to CKD patients at 6 g/day reduces serum indoxyl sulfate [98] by absorb ingindole from the colon. It has been used in Japan since 1991 to delay deterioration of renal function in CKD patients, although the clinical benefit is still unclear [99-101].

A second class of toxins comprises advanced glycation endproducts (AGEs) formed in processed foods via the Maillard reaction [102]. Typical dietary AGEs are Nε-carboxymethyl-lysine and Nε-carboxyethyl-lysine; their serum concentrations correlate with their dietary intake in uremic patients [103,104]. Recent studies report that AST-120 and sevelamer each decrease serum AGE levels in pre-dialysis CKD [105] or hemodialyzed patients [106] and imply that both drugs bind dietary AGEs in the gut. The vast range of toxins and their hypothetical role in renal pathology [107,108] pose a challenge for designing novel, more efficient toxin scavengers – it is unclear where future development should focus. Non-specific binding may be beneficial for sequestering an array of potential toxins; improved binding capacity remains critical to lowering the therapeutic dose.

#### 6.3.6 Treatment of Hyperoxaluria

In its most severe form, excessive urinary oxalate excretion (hyperoxaluria) is caused by a deficiency of the hepatic peroxisomal enzyme alanine-glyoxylate aminotransferase *(Agxt)*. Overproduction of oxalate may lead to recurrent kidney stones, progressive nephrocalcinosis, and loss of kidney function. Oxalate is partially eliminated by the GI tract acting as a sink for systemic oxalate; this effect is particularly pronounced in CKD [109]. An elegant therapeutic approach is to treat orally with crosslinked crystals of oxalate decarboxylase that remain stable in the GI tract and efficiently metabolize oxalate in the gut [110].

### 6.4 Normalization of Bowel Dysfunction

With a 15% prevalence in North America, chronic idiopathic constipation (CIC) is a significant burden on society [111,112]. Normal transit constipation is the most common form, but even within this population, segmental transit time within the colon can be variable [113] and the majority of patients had abnormally low small intestine motility [114]. Irritable bowel syndrome is a common GI disorder associated with alterations in motility, secretion, and visceral sensation. The most common form of IBS is the constipation-dominant form, which causes abdominal pain as well as replicating most of the CIC symptoms.

The intestinal motor secretory reflex controls motility and intestinal fluid content, ultimately driving transit. In the past few years, novel non-systemic approaches have emerged to selectively activate prosecretory pathways. Linaclotide (Ironwood Pharmaceuticals) is a close analog of the heat stable *Escherichia coli* enterotoxin, a potent agonist of guanylate cyclase receptor, which via cGMP production stimulates the cystic fibrosis transmembrane conductance regulator (CFTR) chloride channel. Linaclotide acts locally in the intestinal tract to induce secretion. It has a very short half-life in the GI tract and very low systemic exposure [115]. In multiple studies in CIC patients, linaclotide improved bowel habits and symptoms [116], and in a phase II study demonstrated statistically significant improvementsin abdominal pain and discomfort, as well as bowel normalization [117].

RDX5791 (Ardelyx), a non-systemic NHE3 inhibitor described above, modulates the re-absorption of sodium and fluid in the upper GI and alters stool form [118], as was demonstrated in a multi-ascending dose phase I study in healthy subjects [119]. Both RDX5791 and linaclotide demonstrated a pharmacological effect in a rat model of visceral hypersensitivity [120,121], a hallmark of IBS suggesting that an influx of intestinal fluid in the upper GI tract may elicit a neurohormonal signal mediating local anti-nociception.

Inhibitors of the IBAT reduce active ileal reabsorption of bile acids. This results in an increased concentration of bile acids entering the colon, stimulating colonic motility and secretion. Bile acids induce propulsive contractions in the human colon, and induce secretion through activation of adenylate-cyclase, increased mucosal permeability, and inhibition of apical Cl^-^/OH^-^ exchange. A3309 (Albireo), a selective inhibitor of the IBAT, increases stool frequency and reduced time to first stool. The most common side effects are abdominal pain and diarrhea [122]. Of note, the use of non-absorbable, luminally active dinucleotide mimics, such as EPX-16006 (Epix Pharmaceuticals), to trigger chloride secretion via stimulation of the colonic P2Y2 receptor has been shown to increase feces production and improve gastrointestinal motility in preclinical models [123].

## 7 OPPORTUNITIES FOR NON-SYSTEMIC DRUGS

Non-systemic drugs clearly have earned a place in the pharmacopeia, treating important diseases with remarkably good safety profiles. The field is approaching a critical transition from its prior focus on niche polymeric materials that sequester intestinal small molecules to non-systemic agents targeting transporters and receptors expressed at the intestinal epithelia. Proteomic analysis of membrane proteins lining the GI epithelia, or *luminome*, is in its infancy, but recent studies have revealed a vast repertoire of potential targets [124,125]. Approximately 45 transporters, 20 digestive enzymes, 105 signaling proteins, and 95 proteins were identified from preparations of brush border membranes isolated from murine jejunum. To fully exploit these findings, further work is needed to: i) determine the function and therapeutic potential of those proteins; ii) identify those effectively present at the apical pole; and iii) probe whether a non-absorbed molecule can modulate protein activity.

The remarkably rapid remission of diabetes in patients with Roux-en-Y by-pass surgery [126] has transformed our vision of the roles of these previously unidentified or unexplored hormones, receptors, and factors in the systemic regulation of metabolism [127, 128]. Examples of such receptors are TGR5 [129] and GPR119 [130], G-protein coupled receptors responding to bile acids and dietary lipids respectively, triggering secretion of GLP-1, and peptide YY (PYY) with pleiotropic effect across various pathways to restore insulin sensitivity and induce satiety. Non-systemic agents targeting one or more of these receptors would have a benefit in the treatment of diabetes and metabolic diseases.

Host-gut flora interaction plays an important role in the etiology of some chronic GI disorders.Whether a non-systemic intervention could reverse diseases such as IBD or IBS is yet unclear, but these evolving fields point to intestinal transporters and receptors as possible factors mediating the disease. Protease-activated-receptor 2 (PAR-2) expressed on the apical side of the GI tract are suspected of playing a key role in the pathology of diarrhea-predominate IBS (IBS-D) [131] and IBD [132]. These receptors are activated as a result of abnormal luminal protease activity in IBS-D patients. Colonic PAR-2 stimulation appears to be the primary event in TJ openings that increase GI permeability to flora antigens. Bacterial antigen translocations subsequently trigger an innate immune response that cascades in further submucosal PAR-2 stimulation causing increased intestinal secretion (diarrhea) and nociceptive signaling to afferent neurons (pain). Non-absorbed PAR-2 antagonists could potentially restore mucosal integrity and resolve inflammation symptoms.

In IBD, the role of epithelial sensors to microbial products resulting in increased immune stimulation, epithelial dysfunction, or enhanced mucosal permeability is now well established. Certain epithelial transporters (PepT1) [133,134] and receptors (Toll-like receptors) participate in sensing or translocating bacterial antigens [135] and could therefore constitute novel therapeutic targets. Finallynew safe and effective anti-diarrhea drugs are urgently needed in developing countries: the role of intestinal ion channels (CFTR, CaCC) and ion-sensors (Ca^2+^-sensing receptor [136]) in the etiology of secretory diarrhea and their apical presentation in the gut, make them attractive targets for designing non-absorbable agents with a high therapeutic index [137-139].

## Figures and Tables

**Fig. (1):**
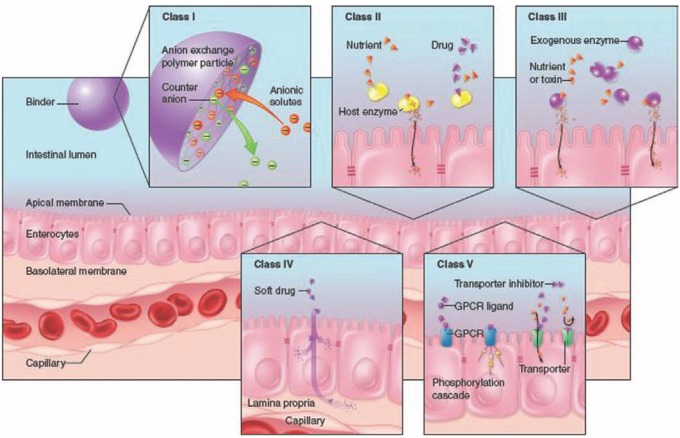
**Mode of action of non-absorbed drugs.** Class I: sequestering agent (exemplified by an anion-exchange polymer); Class II: Ligands of soluble intestinal enzymes; Class III: exogenous enzymes; Class IV: Minimally absorbed and rapidly metabolized drugs (also referred to as *soft* drugs); Class V: Ligands of apical targets. (The modes of action of each class are described in the text)

## ABBREVIATIONS

ADME: = Absorption, distribution, metabolism, and excretion
AGEs: = Advanced glycation end products
CFTR: = Cystic fibrosis transmembrane conductance regulator
CHF: = Congestive heart failure
CIC: = Chronic idiopathic constipation
CKD: = Chronic kidney disease
FGF-23: = Fibroblast growth factor 23
GI: = Gastrointestinal
GLP-1: = Glucagon-like peptide-1
HbA1c: = Glycated hemoglobin A1c
HMG-CoA: = 3-Hydroxy-3-methylglutaryl-coenzyme A
IBAT: = Ileal bile acid transporter
IBD: = Inflammatory bowel disease
IBS: = Irritable bowel syndrome
IBS-D: = Diarrhea-predominate IBS
LDL: = Low-density lipoprotein
LPS: = Lipopolysaccharides
NaP2b: = Sodium/phosphate co-transporter
NHE3: = Na/H antiporter 3
NPC1: = Niemann-Pick C1-Like 1
PAR-2: = Protease-activated-receptor 2
PLA2: = Phospholipase A2
PTH: = Parathyroid hormone
RAAS: = Renin-angiotensin-aldosterone-system
TJ: = Tight junction
tPSA: = Total polar surface area
